# Electro-synthesis of novel nanostructured PEDOT films and their application as catalyst support

**DOI:** 10.1186/1556-276X-6-364

**Published:** 2011-04-27

**Authors:** Cuifeng Zhou, Zongwen Liu, Yushan Yan, Xusheng Du, Yiu-Wing Mai, Simon Ringer

**Affiliations:** 1 Australian Centre for Microscopy and Microanalysis, University of Sydney, Sydney, NSW 2006, Australia; 2 Department of Chemical & Environmental Engineering, University of California, 900 University Avenue, Riverside, CA, USA; 3 School of Aerospace Mechanical & Mechatronic Engineering J07, University of Sydney, Sydney, NSW 2006, Australia

## Abstract

Poly(3,4-ethylenedioxythiophene) (PEDOT) films doped with nitric and chlorine ions have been electrochemically deposited simply by a one-step electrochemical method in an aqueous media in the absence of any surfactant. The fabricated PEDOT films were characterized by scanning electron microscopy, transmission electron microscopy, and Raman spectroscopy. The results indicate that the hierarchical structured PEDOT film doped with nitric ions displays a 'lunar craters' porous morphology consisting of PEDOT nano-sheets with a thickness of less than 2 nm. The effect of counter ions on the electro-polymerization, the electrochemistry, and the morphology of the polymer film was studied. Compared with PEDOT film doped with nitric acid, PEDOT film deposited in the presence of chlorine ions shows irregular morphology and less electrochemical activity. The specific nanostructure of the polymer was further studied as catalyst support for platinum nanoparticles to methanol electro-oxidation.

## Introduction

Poly(3,4-ethylenedioxythiophene) (PEDOT) is an important π-conjugated conducting polymer, which is currently being investigated for use in many fields [1], such as antistatic and anticorrosion materials, artificial muscles, electrode materials in batteries, super-capacitors, display devices, and biosensors. Although various 1D or 2D PEDOT nanomaterials, such as nanofibers, nanospheres, nano-tubes, and nanorods [2-6], have been prepared and studied, there are few reports on the more complicated hierarchical structure of this functional polymer. PEDOT film is one of the main applied forms of this functional material. For example, PEDOT films are recently studied as catalyst support for Pt or Pd nanoparticles for either electro-oxidation of methanol or ethanol [7,8], which can be potentially used in direct methanol fuel cell (DMFC) or sensors to some chemicals, such as nitrite, bromate, oxygen, hydrogen peroxide [9]. As many applications of the PEDOT material are related to its microstructure and electrochemical activity, studies on the film surface control and physical chemical properties are very important.

Electrochemical polymerization is a convenient method to prepare PEDOT, and generally a film yields on the surface of the anode. The electro-synthesis can be done both in organic and aqueous solutions. Due to the low solubility of EDOT in water at room temperature, previous research on the electro-polymerization of PEDOT was usually done in an organic solution, such as acetonitrile and propylene carbonate [10,11]. Recently, there are growing interests in the study of electro-polymerization of PEDOT films in an aqueous solution [12-19]. It is known that the properties of conducting polymers are strongly dependent on their morphologies and their physical and chemical structures. Polypyrrole micro-containers have been synthesized electrochemically by direct oxidation of pyrrole monomer in an aqueous solution [20,21]. Surfactants were often used to control the morphology of the PEDOT film with electrochemical methods recently [12-17]. Sodium dodecylsulphate (SDS) has also been used in the electro-synthesis of PEDOT with different morphologies (globular and fibrous) [12]. PEDOT/PSS (poly(4-styrene sulfonate)) film with microstructures of micro-rings/arrows and bubbles has been prepared with the cyclic voltammetry method and the influences of applied potentials and surfactant (PSS) on the morphology of the resulted film were also investigated [13]. PEDOT micro-cups with diameters in the range of 1 to 4 mm were generated by direct oxidation of EDOT in the aqueous solution of LiClO_4 _and tri (ethylene glycol (EG)) on the ITO electrode with a PSS/PDDA multilayer coating [19].

The conductive polymers, such as PAni or PEDOT have been studied as catalyst or its support for hydrogen and methanol fuel cell applications [22-24]. Pt supported on PAni-based nano-tubes and nanofibers showed an excellent electrochemical activity for methanol oxidation, respectively [23,24]. In this work, a three-dimensional (3D) nanostructured PEDOT was fabricated through a simply one-step electrochemical route in an aqueous solution in the absence of any surfactant. Microscopic studies indicated that the films are comprised of fine electroactive polymer sheets with the edge thickness of less than 2 nm, which is only slightly larger than the well known graphene sheets. Moreover, a lunar crater porous morphology was observed on one surface of the films. To the best of our knowledge, this is the first report concerning the deposition of such nanostructured PEDOT films on Pt and its application as catalyst support for methanol oxidation.

## Experimental

### Electro-deposition of PEDOT

The electrochemical deposition of PEDOT was carried out on a CHI1202 Electrochemical Analyzer (CH Instruments). All solutions were prepared in distilled water and all potentials reported are referenced to saturated calomel electrode (SCE). An electrolyte of 0.1 M KNO_3 _(or 0.1 M KCl) + 0.01 M EDOT was used for the deposition of PEDOT doped with nitric ions (denoted as PEDOT-NO_3_) (or doped with chlorine ions (denoted as PEDOT-Cl)) on platinum electrode. A three-electrode electrochemical cell was used for the electrochemical measurements, where the counter electrode was a Pt foil and the reference electrode was a SCE. The charge consumed was monitored and was considered as a measure of the mass of the deposited PEDOT. The polymer was removed carefully from the electrode and dispersed in EG under ultrasonic irradiation to prepare the PEDOT suspensions.

### Electrocatalyst fabrication and the methanol oxidation

Pt particles in EG were prepared in situ by a microwave assisted EG reduction method. 0.1 g H_2_PtCl_6 _and 0.1 g NaOH were dissolved in 5 mL EG. The glass bottle was placed in the center of a microwave oven and heated for 13 s to prepare the Pt nanoparticle containing solution. The fabricated black solution was very stable and was mix with the above PEDOT suspensions. Seventy microliters of the Pt containing solution was added dropwise to the above PEDOT suspension with strong ultrasonic irradiation to prepare the electrocatalyst composites. After centrifugation and washing with ethanol, the material was dispersed in 0.05 wt% Nafion solutions and sonicated for 20 min to prepare the catalyst ink. Eight microliters of such catalyst solution was added to the surface of the glass carbon (GC) electrode (Φ = 3 mm) and dried in air. Prior to the electrochemical measurement, the catalyst covered electrode was soaked in the electrolyte solution (1 M CH_3_OH + 0.5 M H_2_SO_4_) for 10 min.

### Morphology and spectrum of the materials

Transmission electron microscopy (TEM) characterization was performed on a Philips CM12 operating at an accelerated voltage of 120 kV and scanning electron microscopy (SEM) analyses were performed on a Zeiss ULTRA plus. Raman spectroscopy of the products was performed on an Invia Renishaw Raman using a He-Ne laser at 633 nm wavelength.

## Results and discussion

### Electropolymerization of PEDOT

The conductive polymers are always prepared in their oxidation form with doping ions. The doping ions in the PEDOT have been reported to have great influence on the electrical properties of the film [12,13]. For example, the PEDOT films doped with Cl^- ^have much less electrical conductivity than those doped with NO_3_^- ^[12]. Here we studied the effect of the doping ions (Cl^- ^and NO_3_^-^) on the electro-polymerization behavior, the morphology, and the electrochemical properties of the resulted PEDOT polymer film.

**Figure 1:**
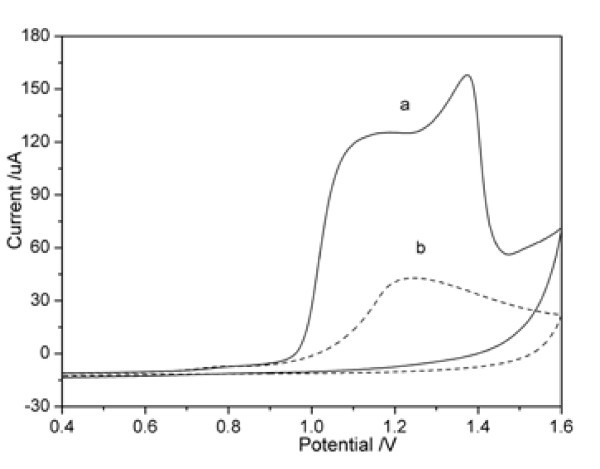
**Cyclic voltammograms of Pt in 0.01 M EDOT + 0.1 M KNO**_**3 **_**and 0.01 M EDOT + 0.1 M KCl**. Scan rate: 50 mV/s.

Figure 1 shows the cyclic voltammograms of the electrochemical oxidation of EDOT on the Pt electrode with Cl^- ^and NO_3_^- ^as doping ions, respectively. In the case of nitric ions (Figure 1a), two oxidation peaks appeared at about 1.15 and approx. 1.37 V. Similar to the previous report [18], the first peak can be attributed to the oxidation of the EDOT and the second peak may be related to the over-oxidation of the preformed PEDOT or its oligomers. However, in the case of chlorine ions (Figure 1b), only a poorly resolved current peak at 1.25 V appeared. The current is much less than the one with nitric ions, although the other parameters are the same. The electro-polymerization of the EDOT in the presence of chlorine ions is quite different from that previously reported in the presence of perchlorate ions [18] and dodecylsulphate anions [12]. Obviously the electrochemical process of the electro-deposition of PEDOT doped with chlorine ions needs further investigation.

**Figure 2:**
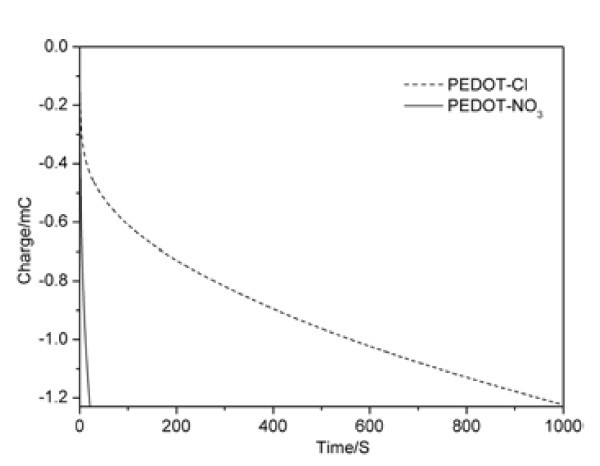
**Chronocoulemetry of PEDOT deposition on Pt in 0.01 M EDOT + 0.1 M KNO**_**3 **_**and 0.01 M EDOT + 0.1 M KCl**.

**Figure 3:**
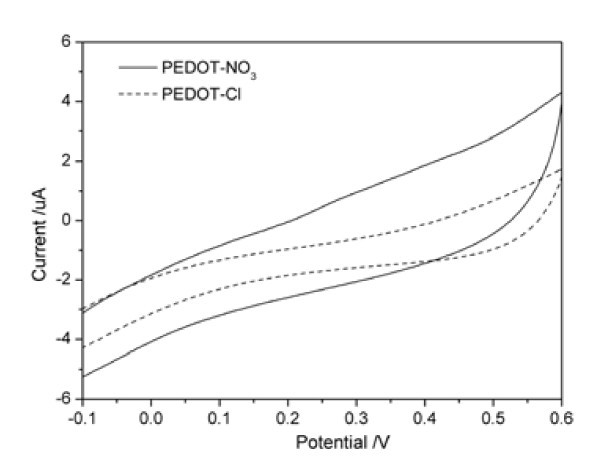
**Cyclic voltammograms in the phosphate buffer solution**. Scan rate: 50 mV/s. **(a)**PEDOT-NO_3 _and **(b)**PEDOT-Cl.

PEDOT films doped with chlorine and nitric ions were electro-synthesized with the chronocoulometry method, as shown in Figure 2. As the charges consumed implied the amount of the electrical conductive polymer on the electrode, the same charge was consumed to fabricate the same amount of polymer doped with different ions. The charges were consumed more quickly in the presence of nitric ions than those in the presence of chlorine ions, indicating the faster deposition of the PEDOT doped with nitric ions. With the electro-polymerization proceeding, a homogeneous deep blue polymer film doped with nitric ions was deposited on the Pt electrode surface. Different from the color of the PEDOT-NO_3_, PEDOT-Cl film is brown. The electrochemical behaviors of the PEDOT films with different doping ions were also investigated. As shown in Figure 3, the current of PEDOT-Cl is much less than that of PEDOT-NO_3_, indicating that the PEDOT doped with nitric ions is more electro-active than those doped with chlorine ions, which is in consistence with the above CV and chronocoulometry results.

**Figure 4:**
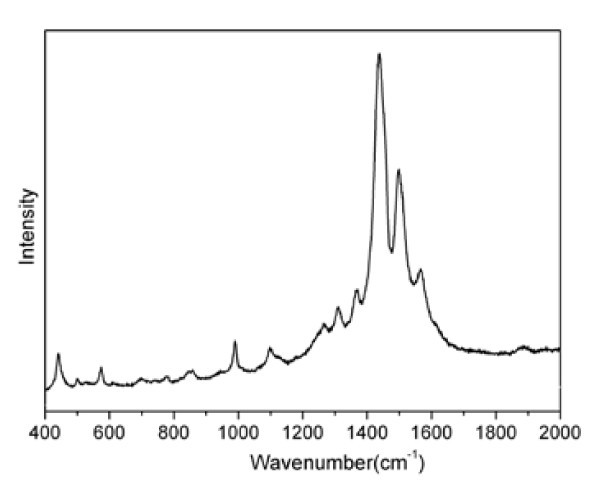
**Raman spectrum of PEDOT-NO**_**3 **_**film**.

Raman studies were carried out to further determine the vibration modes of the films in the wavenumber range of 400 to 2000 cm^-1^, as shown in Figure 4. The peaks at 1568 and 1500 cm^-1 ^are originated from the asymmetrical stretching of C_α _= C_β_, and the most intense peak at 1438 cm^-1 ^can be attributed to the symmetrical stretching of C_α _= C_β _(in the monomer EDOT, C_α _is the carbon atoms connecting with the sulfur atom, while C_β _is the carbon atoms connecting with C_α_). The peaks at 1366 cm^-1 ^are assigned to the C_β"_-C_β" _inter-ring stretching, and the peaks at 1267 cm^-1 ^are assigned to the C-C inter-ring stretching. The peaks at 1100 and 697 cm^-1 ^are assigned to the C-O-C stretching and C-S-C bending, respectively. A peak from C-C anti-symmetrical stretching mode can be seen at 990 cm^-1^. The resulted spectrum is just similar to those of other results and the absence of any signal in the 650 to 680 cm^-1 ^region of the spectrum could lead to similar conclusion that the electrodeposited PEDOT chains has a highly planar structure [11,16,25].

**Figure 5:**
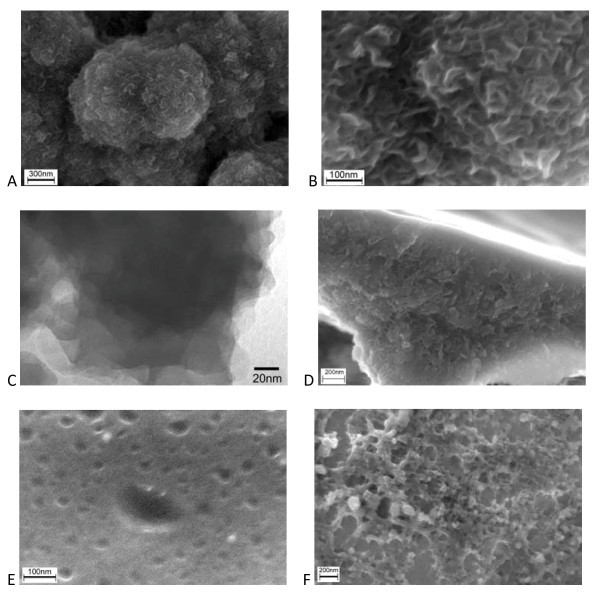
**SEM and TEM images of the PEDOT-NO**_**3 **_**film**. **(a, b)**up-surface; **(c) **the cross section and **(d) **the back-surface; **(e) **TEM image; **(f) **SEM images of PEDOT-Cl film.

Based on the above results, the electroactive PEDOT film doped with nitric ions was selected as the catalyst support. The SEM images of the PEDOT film are presented in Figure 5a, b. The polymer film consists of flower-like particles, each comprising of many fine polymer sheets. In the cross-section image of the film (Figure 5c), the sheets seem to connect with each other, leaving countless irregular pores in the film. The thickness of the nanosheets is always less than 10 nm, which is also confirmed by TEM observation (Figure 5d), in which even approx. 2-nm sheet thickness can be identified from the folded edge of the polymer sheets. Due to their high aspect ratio, the nanosheets generally scrolled and bent to minimize the surface energy, similar to the behavior of the graphene nanosheets [26]. On the other side of the film facing to the Pt substrate (Figure 5e), many pores with nanoscale size can be found, which is just like the lunar craters. It should be noted that such nanostructure is not common in the electrodeposited PEDOT films, as no such sheet-like structure was found in electropolymerized PEDOT-Cl materials (Figure 5f).

### Methanol oxidation on Pt-PEDOT catalyst

**Figure 6:**
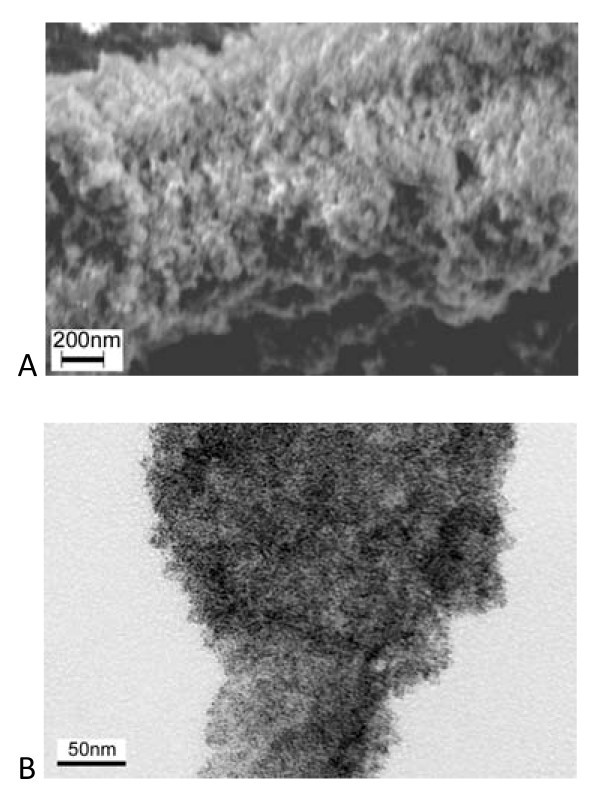
**Images of the Pt-PEDOT composites**. **(a)**SEM and **(b)**TEM.

Figure 6 shows the morphology of the Pt-PEDOT materials. The white grains in the SEM micrographs (Figure 6a) are attributed to platinum nanoparticles. Due to high degree of porosity nanosheet structure of the PEDOT film, it favors for platinum particles to be uniformly dispersed over the film surface. The EDS results also prove the existence of Pt in the composite and the content of the platinum in the composite was 50 wt%. In the TEM image (Figure 6b), the Pt particles were uniformly dispersed on the surface and edge of the PEDOT sheets. Some aggregates with a diameter of about 20 to 60 nm can also be observed occasionally and they are composed of fine Pt particles (about 3 to 6 nm of size).

**Figure 7:**
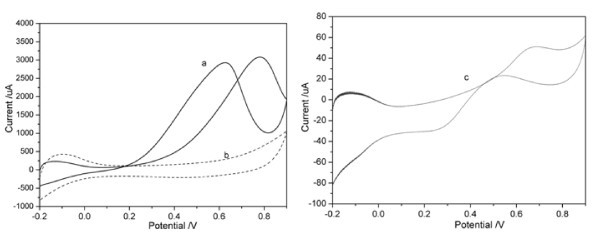
**Cyclic voltammograms of (a) Pt-PEDOT/GC and (c) Pt disk electrode in 1 M CH**_**3**_**OH + 0.5 M H**_**2**_**SO**_**4**_**, and (b) Pt-PEDOT/GC in 0.5 M H**_**2**_**SO**_**4 **_. Scan rate: 100 mV/s.

The cyclic voltammogram of a Pt-PEDOT/GC electrode in 0.5 M H_2_SO_4 _at a sweep rate of 100 mV/s is shown in curve b in Figure 7. The current peaks appearing between -0.2 and 0.0 V are due to adsorption and desorption of hydrogen atoms on the Pt surface. The CV is similar to those of Pt metal [7], suggesting that Pt-PEDOT film is electrochemically active. The curve a in Figure 7 illustrates the electrocatalytic activity of the Pt-PEDOT/GC electrode toward oxidation of methanol. Comparing with the curve b, two new peaks appeared above 0.15 V in curve a in Figure 7, in which the forward current peak is attributed to the oxidation of CH_3_OH molecule and the backward current peak to the oxidation of adsorbed intermediates. The current peaks related to hydrogen adsorption in curve a in Figure 7 are much less than the ones in curve b, indicating the adsorption of some reaction intermediates from the oxidation of methanol on the Pt nano-catalysts. Obviously, the methanol oxidation current on the Pt-PEDOT catalyst (curve b) has been enhanced by more than 70 times than that of Pt electrode (curve c), exhibiting the advantage of the application of nanostructured PEDOT catalyst support and Pt nanocatalyst. The higher current for the methanol oxidation on Pt-PEDOT catalyst indicates their higher electrocatalystic activity of the novel catalysts, which is believed to be related to their porous nanostructure and high electroactivity.

## Conclusion

PEDOT films with a flower-like nanostructure on Pt electrodes have been electrochemically generated simply by a one-step electrochemical method in an aqueous media in the absence of any surfactant. Inorganic counter ions have great effect on the electrochemistry and morphology of the electropolymerized PEDOT films. The morphology of the prepared PEDOT film doped with nitric ions shows porous hierarchical nanostructure based on fine nanosheets which develop large specific surface area with high electrochemical activity. Electrocatalysts comprised the PEDOT film and Pt nanoparticles showed high catalyst performance to methanol electro-oxidation.

## Abbreviations

DMFC: direct methanol fuel cell; EG: ethylene glycol; GC: glass carbon; PEDOT: poly(3,4-ethylenedioxythiophene); PSS: poly(4-styrene sulfonate); SEM: scanning electron microscopy; SCE: saturated calomel electrode; SDS: sodium dodecylsulphate; TEM: transmission electron microscopy.

## Competing interests

The authors declare that they have no competing interests.

## Authors' contributions

CZ carried out the electrocatalyst study and morphology characterization, participated in the sequence data analysis and drafted the manuscript. ZL and YY conceived this study. ZL and SPR financial supported for the spectrum experiments and involved in revising the manuscript. XD conceived the study, carried out the electrodeposit of PEDOT, participated in the design of the study, performed the statistical analysis, drafted and involved in revising the manuscript critically for important intellectual content. YWM conceived this study and involved in revising the manuscript. All authors read and approved the final manuscript.
